# The Existence of a Phthisical Contagium

**Published:** 1891-07-11

**Authors:** 


### THE PRACTITIONER'S MIRROR AND RETROSPECT.

[*The Editor will be glad to receive offers of co-operation and contributions from members of the profession. All letters should be addressed to* THE EDITOR, THE LODGE, PORCHESTER SQUARE, LONDON, W.]

### MEDICINE.

### THE EXISTENCE OF A PHTHISICAL CONTAGIUM.

It has been said that the discovery of the bacillus has been a direct loss, since it has withdrawn men from the study of those conditions of health which render futile the attacks of such invaders. We had learned how such health might often be attained, but we have not learned how to kill our bacillus. It is clear that while we are perfectly uncertain whether we shall ever be able to attack the bacillus directly in the tissues, our present duty is to render human beings able to resist its attacks. In addition to this we must determine whether the disease is contagious, and if so how we may stop the possibility of infection. From the time of Galen the question of the contagiousness of phthisis has been discussed, and even the epoch-making discoveries of Koch have not enabled us to give a definite reply from the practical point of view. The evidence for possible and occasional contagion is absolutely certain, and, indeed, the proofs of such an origin for ordinary cases of phthisis seem daily to grow more overwhelming.

Still, as a recent writer puts it, there is most probably a "something as yet unexplained which forms an important factor, without which the cause of the incidence of the disease on particular localities and individuals cannot be definitely arrived at."

After the discovery of bacilli in 1882, it was gradually proved that they are always present in cases of phthisis and tubercular affections, and not in diseases where such lesions are absent. Next it was shown that they could be cultivated apart, and finally that such pure cultivations either by inoculation or in the form of spray could reproduce the disease in many animals with practically certain results. Instances of the accidental infection of human beings have been also noted again and again. Thus a very robust laboratory attendant had been warned of the great danger of venturing near a spray by which cultivations of the bacilli were scattered among a number of animals, all of whom, by the way, became rapidly tuberculous. He disregarded the warnings and fell a victim to his rashness.

The present evidence goes to prove that the disease is usually communicated by germs carried in dried particles of sputa which are inhaled by other individuals. But the wonder is that we are not all infected by the almost universal presence of these germs. They do not multiply indeed outside the body, but their spores retain their vitality till inhaled perhaps months after they left the lung where they were formed. In some countries where the germ has once been introduced almost every individual does become affected. This was the case with the South Sea Islands, in which phthisis was unknown before it was brought by Europeans. Yet in England and Wales we find nothing of the kind. There were 44,700 deaths from phthisis indeed in 1889, but all these centres of infection have failed to render the greater part of the population tuberculous, although it is calculated that each patient may give off 20 millions bacilli daily.

In the first place it has been assumed that all who escape are protected by a sort of vaccination just as we are freed from small pox or from second attacks of scarlet fever, but this is certainly untrue if taken in the ordinary sense. For individuals who become affected, like the laboratory assistant above-mentioned, when exposed to powerful infection, and the animals, such as guinea pigs, which are so easily killed by inoculation with phthisical sputa, are certainly not in a protected state. Moreover, men develop phthisis at almost every age, showing either that they were unprotected, or that the protection had worn out, or else that there is no such thing as infection.

If we look into the question a little more closely we find that there are natural impediments to infection. Over the greater part of the free surfaces of the body the epithelium acts as a natural shield, and most tubercular lesions commence in the neighbourhood of wounds or of certain parts of the mucous membranes, notably in the lungs and intestinal tube. We find, too, that the spores take some time to germinate, and if removed before ten days or a fortnight, leave no descendants behind them. Now, there is constant removal of foreign bodies from the air-tubes by the ciliated cells, and by coughing; moreover, their entrance is made extremely difficult by the beautiful filters through which the air is conducted. Solid particles of carbon do, indeed, enter and remain in the lungs of miners in large quantities, but there is reason to think that bacilli and their spores are expelled more easily. We see, too, that these tubercular lesions usually commence just where the expulsive action is deficient, as in the apices of the lung, and in lungs with imperfect expansion. A second line of natural defence is provided by the activity of the tissues themselves. Whether it be the blood serum, or the white cells, as Metschnikoff declares, which destroy the invaders, one thing is certain, that relatively large numbers of injurious germs are thus destroyed before they have time to effect a permanent lodgment. If these spores do enter by wounds or past the filters and ciliæ they have to face the struggle with the tissues themselves. The essence of Koch's recent remedies lies in this, that some of the products of the bacilli stir up to greater activity the natural defenders of the body, by whom they are, if not at once killed, at least cut off from food and communication with the rest of the tissues. Two things are clearly of importance, the number and vitality of the invaders on the one hand, and the number and vitality of the defenders on the other. Now we are apt to lose sight of the importance of the number of the invading organisms. Experimentally, Bollinger proved that less than 800,000 bacilli injected into the peritoneum usually fail to cause disease. The same fact is shown by the safety found without the use of the spray in aseptic operations done in fairly pure air, and also in the use, by Lawson Tait, of unsterilised but clean water for flushing the abdomen. The few germs thus introduced are quickly destroyed with little or no risk. On the other hand phthisis becomes rampant where the quantity of germs in the air is great. Thus its fatality increases with the herding together of the population, and among those who live in ill-ventilated rooms. It is rarer in outdoor labourers exposed to cold and wet than among their wives. It committed terrible ravages among the pick of the English army until their barracks were better ventilated, and its special haunts in Manchester and other great cities are in closed-up courts and back-to-back houses, where through currents of air do not exist. Again, when patients lie in ill-ventilated rooms, relapses and attacks in fresh parts of the lungs seem to be often due to self-infection from the inhalation of the dust of the dried sputa. These various cases can hardly all be put down to diminished vitality of the persons attacked. The mortality of nurses and house surgeons in consumptive hospitals has been the subject of so much conflicting evidence that it can hardly be brought in evidence, but it is abundantly clear that the disease is most common in places where there is an accumulation of germs in dirty, unventilated, crowded dwellings.

We do not yet know much of the facts which affect the vitality of the invaders, but two conditions exist in which phthisis makes little headway, and which may operate through this diminished vitality of the spores. In high Alpine altitudes, 5,000 feet above the sea level, there appears to be no spread of phthisis among healthy individuals, even though great numbers of patients are collected together. This may be partly owing to the increased expulsive action of the lung in rarefied air, and partly to the feeble activity of the bacilli themselves. Again, as we shall see later on, where the subsoil is drained an enormous diminution of phthisis has occurred in a great number of towns. Sunlight has been proved to have a powerful effect in attenuating and killing these and similar microbes. Ransome and others have shown that light, fresh air, and the presence of dry sandy soil arrest the virulence of the tubercle bacillus. The influence of solar rays and oxygen together, apart from the effects of temperature have, according to Dr. Downes, a very rapidly destructive effect even on those germs which are most hardy. (*The Practitioner*, February, 1891.) "Cultures of the tubercle bacilli," said Koch, "die in five to seven days if exposed at the window in compact masses."

Much stress has of late been laid on dust as a germ carrier. It is found that in a room without currents of air, the germs in a few hours fall to the ground and the air is quite free from them. The Berlin surgeons, by operating in empty rooms with damp floors (from which particles do not ascend) and with previous perfect sterilisation of hands, clothes, instruments, &c., are able to dispense with antiseptics. But carelessly exposed sputa soon dry and give off huge quantities at least of the spores of bacilli. Hence, the enormous importance of its destruction by fire, steam, or the stronger disinfectants. Handkerchiefs and spittoons may fill the air with germs, and the disbelievers in infection ask why there is so little proof of its occurrence in the rooms of the well ventilated houses of the rich if spores are so easily carried by the wind. The difficulty seems to us a real one, though the above-mentioned oxidation and the natural protectors of the body may possibly account for the facts.

On the other hand there is much evidence that houses inhabited by consumptives retain the infection. Cornet showed that the germs adhere to plaster, and it is becoming the custom to disinfect houses where patients have died, as well as their clothes and bedding. In view of the vast expense, misery, and discomfort of carrying out a system of isolation, it becomes of urgent importance to show absolutely that the apparent infection is real and not due to the depressing influence of small, dark, or dirty rooms on the inhabitants.

Infection is also alleged to take place largely in the intestinal tract by the consumption of tuberculous milk, or milk which has been exposed to tuberculous dust, or again from eating tuberculous meat. Here again the financial importance of a right decision is enormous. Experimentally such infection has been proved perfectly possible, and the spores survive the destructive action of gastric juice. Thoroughly boiled milk is no doubt safe, but does ordinary cooking heat the middle of large joints to a point of security?

Supposing that we consider that precautions are here necessary, we are met by a further difficulty arising from the quantity of germs necessary for infection. May we not all be living in a protected state of a minor degree, that is, so accustomed to the attacks of our enemy that we are able to beat back the daily invasion of a small number, just as Pasteur's weaker inoculation would protect sheep from mild anthrax infection, but not from a virulent one? If, therefore, we succeed at vast expense in removing all ordinary channels of infection, shall we not lose even this degree of protection, and fall victims to the first stray germ which enters our lungs or gullet? Such was possibly the case with the South Sea Islanders; and we may ask, Is there any real safety between the destruction of every single germ, and rendering ourselves immune in the highest degree? If 45,000 people are annually killed when 1,000 bacilli are (let us say) necessary for each infection, would the deaths be less or more if the germs were fewer but our susceptibility greater?

It would seem, however, that our degree of immunity is largely inherited, and, therefore, it would be lost very slowly if at all. It is conceivable, no doubt, that the daily imbibition of so many germs affords just the necessary stimulus to enable us to overcome the attacks of to-morrow, and such a theory gains some support from recent discoveries of protective ptomaines, but it certainly is not proven. Our immunity is inherited, otherwise the South Sea Islanders might have acquired it quickly enough, and the inhabitants of high altitudes do not always or necessarily fall victims when they descend to the plains and encounter unusually great numbers of bacilli. It would seem, then, that our practical duty is, on the one hand, to lessen the numbers and force of the invaders by destroying at least all sputa, by boiling milk, thoroughly cooking meat, by living in light, well-ventilated dwellings, and by preventing over-crowding and the erection of houses on damp undrained soils. On the other hand, we must perfect our immunity by aiming at the greatest possible vigour by good food, exercise, and rest, and in every way strengthening the powers of resistance in those exposed to attack; while to the scientist remains the arduous search for some method by which the great discoveries of Koch and his allies may be completed, until an absolute immunity or an absolute remedy be attained.

The evidence for the frequent occurrence of infection is too strong for us to neglect precautions, yet the need of strengthening the resisting forces of our patients is as great as ever, and we urgently need a more complete knowledge of those factors which favour or diminish the activity of the infection.

